# Treatment of infected non-unions with segmental defects with a rail fixation system

**DOI:** 10.1007/s11751-017-0278-6

**Published:** 2017-02-24

**Authors:** Srikanth Mudiganty, Arup Kumar Daolagupu, Arun Kumar Sipani, Satyendra Kumar Das, Arijit Dhar, Parag Jyoti Gogoi

**Affiliations:** 1 Department of Orthopaedics, Kasturba Medical College (Manipal University), Mangalore, Karnataka 575001 India; 2 Department of Orthopaedics, Silchar Medical College, Silchar, Assam 788014 India; 3 Department of Orthopaedics, Tezpur Medical College, Tezpur, Assam 784010 India

**Keywords:** Non-union, Femur, Tibia, Corticotomy, Bone transport, Rail fixation

## Abstract

We conducted this study to evaluate the use of rail fixation system in infected gap non-union of femur and tibia as an alternative to the established Ilizarov circular fixator technique. Prospective study. The study was done in the Department of Orthopaedic surgery in a medical school and level I trauma center to which the authors are/were affiliated. Between June 2010 and June 2015, 40 patients with infected gap non-union of femur and tibia were treated with the rail fixation system. Patients who were willing to undergo surgery and participate in the post-operative rehabilitation were included in the study. After radical debridement, the system was applied and corticotomy done. For closure of bone gap, acute docking and distraction was done in 18 cases and segmental bone transport in 22 cases. Early mobilization of patient was done along with aggressive physiotherapy. Bone and functional results were calculated according to ASAMI scoring system, and complications were classified according to Paley classification. The mean follow-up period was 22.56 months (range 8–44). Bone union with eradication of infection was achieved in all but 1 case (97.5%). Bone results were excellent in 57.5%, good 40%, fair 0% and poor in 2.5% cases, while functional result was excellent in 32.5%, good 65%, fair 0% and poor in 2.5% cases. The rail fixation system is an excellent alternative method to treat infected gap non-union of femur and tibia. It is simple, easy to use and patient-friendly.

## Introduction

Segmental defects created after debridement for infected non-union of long bones can be managed by external fixation and bone grafting, bone transport or microvascular free-tissue transfer [1–4]. The Ilizarov principles are the basis for modern successful treatment [5, 6]. However, when the principles are applied using the conventional Ilizarov fixator with its transosseous tensioned wires, there are disadvantages: local anatomy is distorted; soft tissues are transfixed; there is a risk of neurovascular impingement; and the circumferential fixator is poorly tolerated [7, 12]. The circular fixator is cumbersome and interferes with conduct of personal hygiene, especially when rings are used in upper thigh [8–10]. To overcome these disadvantages, some modifications were introduced, e.g. use of half pins and femoral arches [10, 11]. In contrast, a monolateral external fixator has advantages with being located to one side of the limb, is usually easier to apply and remove and has greater patient acceptance [13, 14]. This study evaluates the use of a monolateral fixator for treating segmental defects after treatment of infected non-unions of the femur and tibia.

## Patients and methods

A total of 40 patients with infected non-unions of the femur and tibia were treated with a monolateral fixator between June 2010 and June 2015. A retrospective review was conducted. The inclusion criteria were all cases of infected non-union of femur and tibia that had a segmental defect; limbs had to be normal in the neurological and vascular status and the contralateral limb functionally good enough so as not to influence the rehabilitation process. Patients who were medically unfit for surgery and those unwilling to participate in the prolonged post-operative rehabilitation were excluded.

Of the 40 patients, the femur was involved in 22 and the tibia in 18. There were 35 males with an average age of 29.3 years and 5 females with average age of 38.3 years. The right extremity was involved in 25 cases and the left in 15. Clinical and radiological examination established the diagnosis. The causes of infected non-union were: sequel to open fractures (19 cases—47.5%); closed fracture treated with internal fixation with subsequent infection (10 cases—25%); and chronic osteomyelitis as a sequel to acute osteomyelitis (11 cases—27.5%). The non-union was classified according to the classification by Rosen as active (3 cases), draining (11 cases) and quiescent (26 cases) [15]. The mean number of previous surgeries was 1.44 (range 0–3) including repeated debridement, intramedullary nailing, plating and/or external fixation.

Treatment consisted of removal of existing implants, radical debridement and the application of the monolateral fixator. Bone debridement continued until residual bone showed evidence of punctate cortical bleeding (the paprika sign). The monolateral fixator was placed laterally in the femur and anteromedially in the tibia. In those cases where a segmental defect of less than 5 cm in the femur (11 patients) or 3 cm in the tibia (7 patients) was encountered, acute docking and subsequent lengthening was carried out. In the remaining cases, segmental bone transport was done. Iliac crest bone graft was added to the docking site in 5 cases of acute docking.

Active and passive mobilization of the adjacent joints was started on second post-operative day. Ambulation and partial weight-bearing was started on the 2nd or 3rd post-operative day depending on pain, bone quality and patient compliance. Distraction at the corticotomy site was started between the 7th and 14th day at rate of 1 mm/day (0.25 mm every 6 h). Pin track dressings were of sterile gauze and normal saline. At the time of discharge, patients were educated to deliver pin track care, perform distraction and physiotherapy for joint mobilization and muscle strengthening. Regular follow-up every 4 weeks was done. Checks on pin loosening, pin track infections were carried out and radiographs obtained. The fixator was dynamised once clinical (the absence of deformity or abnormal mobility, the ability to walk and stand on the operated leg without pain) and radiological (3 or more cortices on two orthogonal views) union was declared [16]. After dynamisation, if the patient could weight bear without pain, the fixator was removed as an outpatient procedure under sedation. The operated extremity was protected with functional cast brace for 4 weeks after removal of the fixator.

Institutional ethics committee approval was obtained for the study. All the patients or their guardians provided informed written consent.

## Results

All 40 patients were available for follow-up. The bone and functional results were graded as excellent, good, fair and poor [17]. (Table 1) The external fixation index (EFI) was calculated as a ratio of the number of days the frame was used to the length of regenerated bone (cm). The mean follow-up period was 22.92 months (range 12–44). The fixator was used for an average of 13.6 months (range 6–30). The mean length of regenerated bone was 7.17 cm (range 1–15). The mean EFI was 56.9 days/cm. The mean residual limb length discrepancy was 1.04 cm (range 0–4).

**Table 1:** ASAMI scoring system [17]

	Bone results	Functional results
Excellent	Union No infection Deformity <7 degree Limb length inequality <2.5 cm	Active, no limp, minimum stiffness (loss of <15 degree knee extension/<15 degree dorsiflexion of ankle), no RSD, insignificant pain
Good	Union with any two of the following: Absence of infection, <7 degree deformity and limb length inequality of <2.5 cm	Active with one or two of the following: limp, stiffness, RSD, significant pain
Fair	Union with only one of the following: Absence of infection, deformity <7 degree, limb length inequality of <2.5 cm	Active with three or all of the following: limp, stiffness, RSD, significant pain
Poor	Non-union/refracture/union + infection + deformity >7 degree + limb length inequality >2.5 cm	Inactive (unemployment or inability to return to daily activities because of injury)
Failures	–	Amputation

*RSD* reflex sympathetic dystrophy

**Fig. 1:**
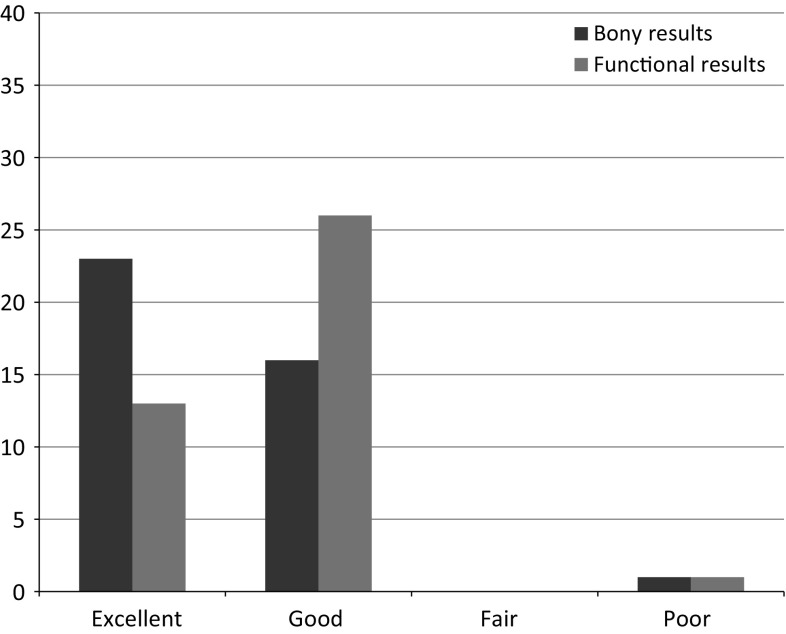
Results

Of the 40 cases included in the study, 23 had excellent, 16 good, none had fair and 1 case had a poor bone result. Functional results were found excellent in 13 cases, good in 26, fair in none and poor in one. The patient with a poor result was a 65-year-old diabetic who had an infected non-union of the mid-shaft of the tibia after an open fracture 13 years previously. This was treated with monolateral frame and bone transport. This resulted in a docking site non-union; he refused secondary bone grafting. The frame was removed and patient opted to walk with crutches indefinitely (Fig. 1).

**Fig. 2:**
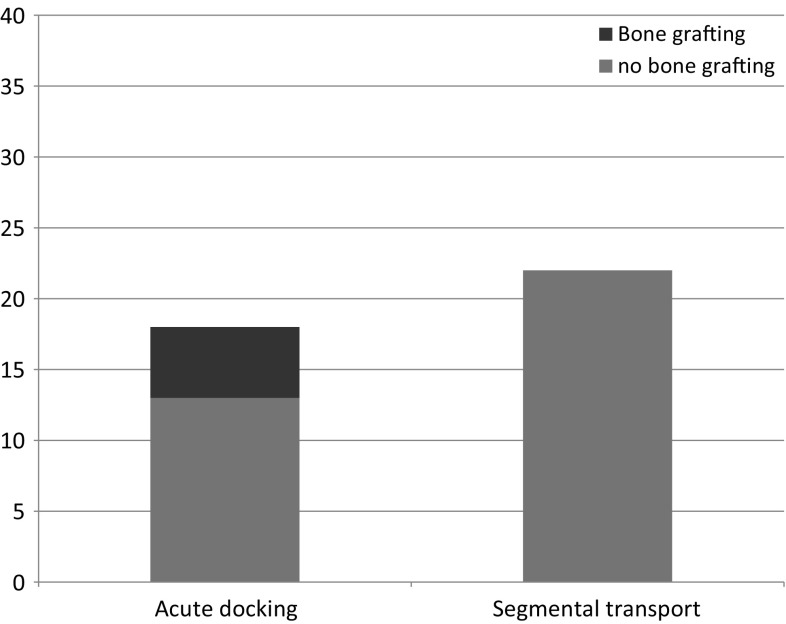
Method of treatment

**Fig. 3:**
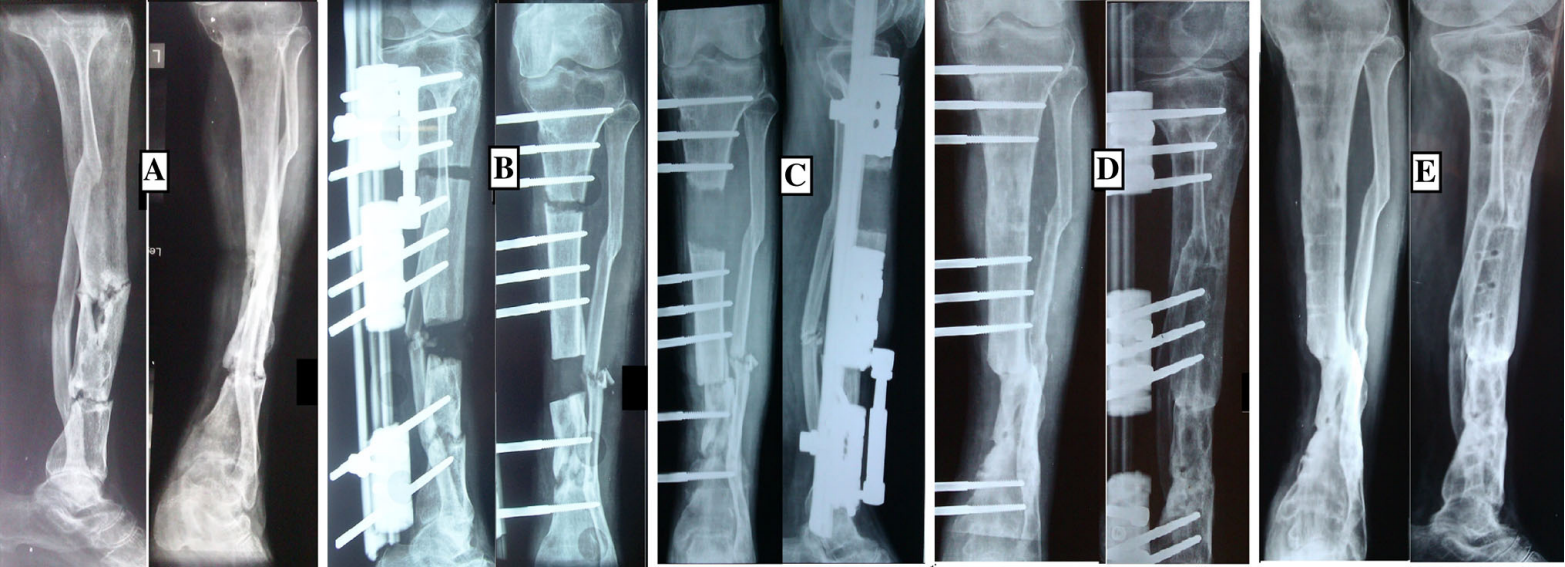
**a**. 35-year-old male with infected non-union of Tibia of 11-year duration following compound fracture of both bone leg. **b** Debridement, application of rail fixation and distraction for 5 days. **c** Callus formation, **d** Consolidation of callus and union at docking site. **e** After removal of the system

**Fig. 4:**
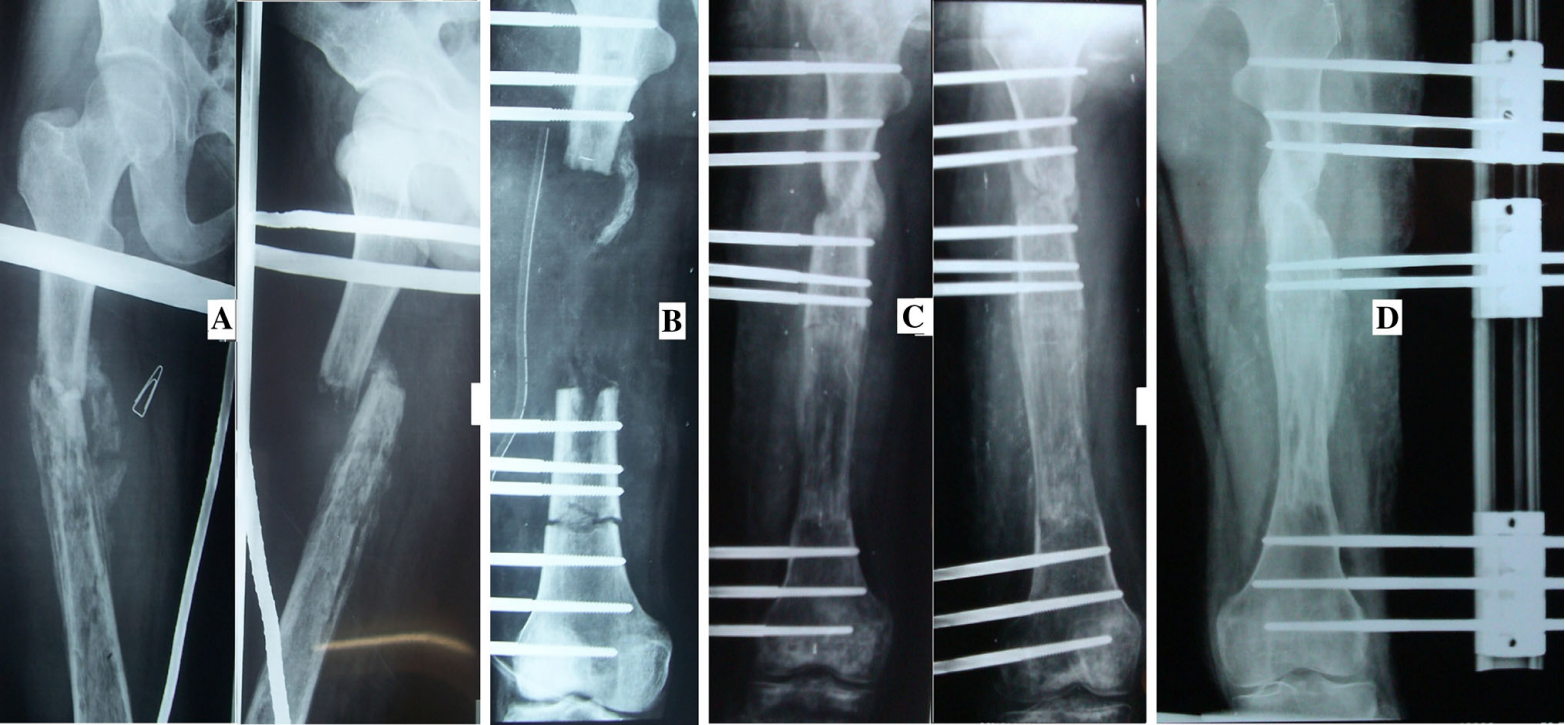
**a** 19-year-old male with infected non-union of femur following pathological fracture due to chronic osteomyelitis. **b** Debridement and rail fixation application. **c** Callus formation. **d** Consolidation of callus and union at docking site (just before system removal)

**Fig. 5:**
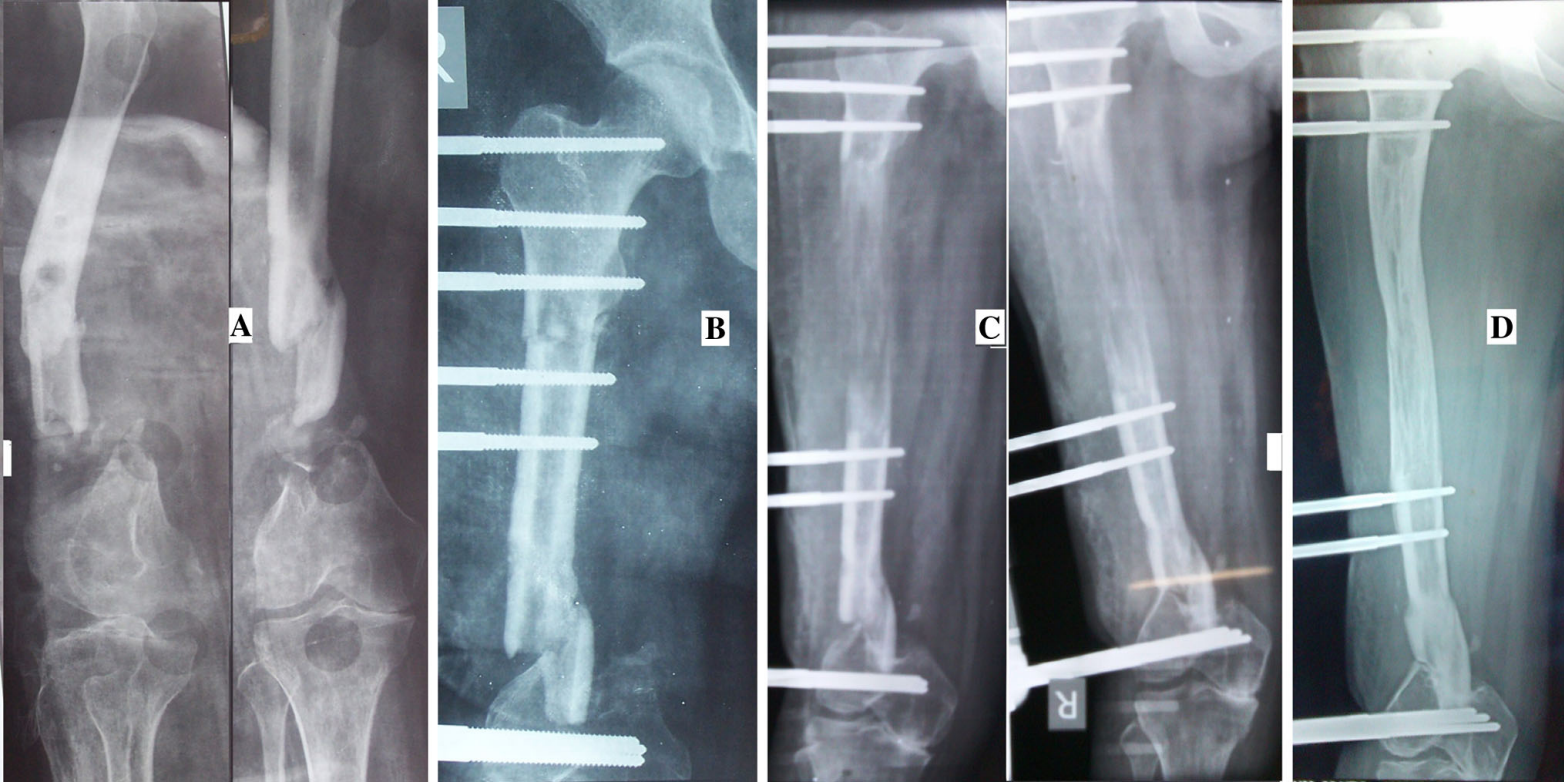
**a** 35-year-old female with infected non-union of femur following compound fracture of Femur with bone loss. **b** Debridement and rail fixation application. **c** Callus formation. **d** Consolidation and union at docking site (just be removal of the system)

**Fig. 6:**
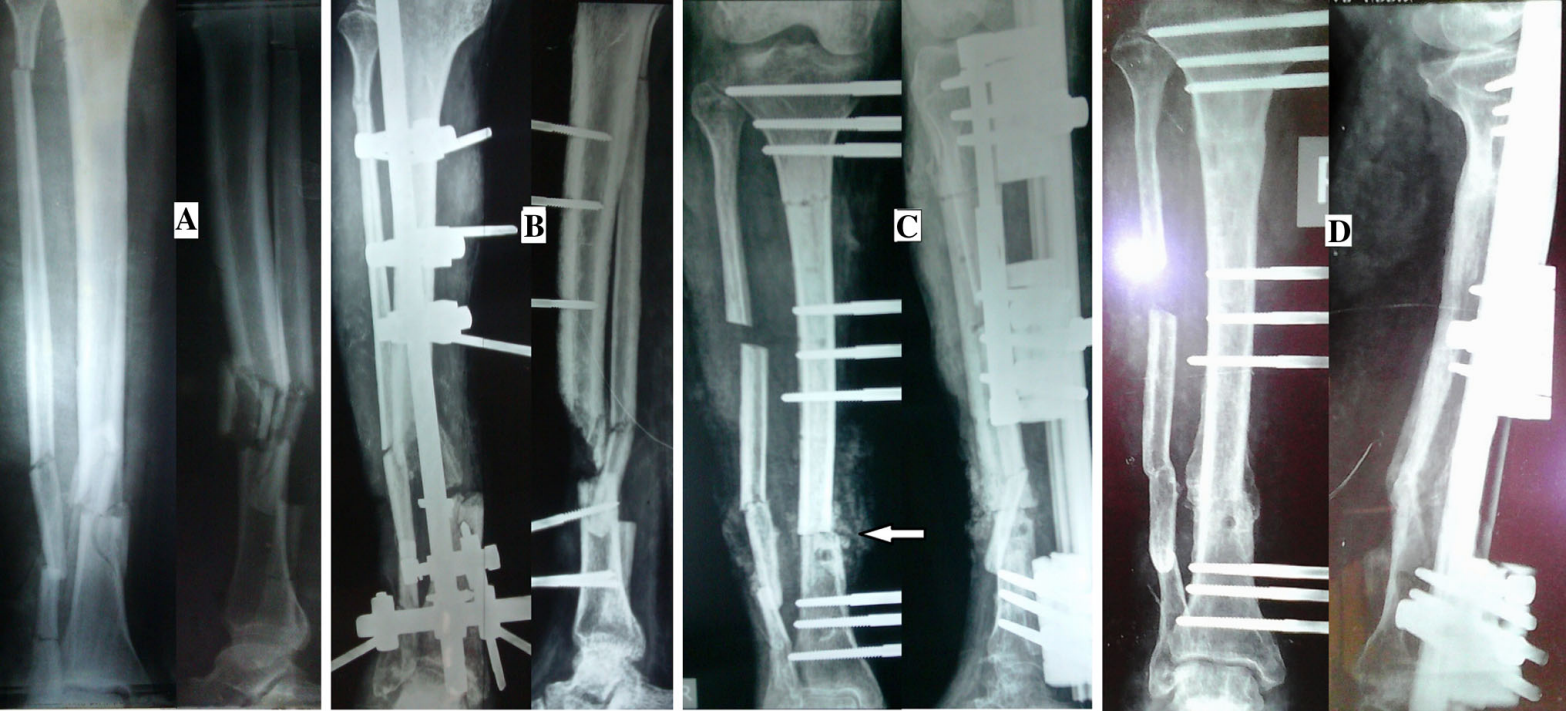
**a** 45-year-old male with compound fracture of both bone leg. **b** Debridement and external fixation. **c** Debridement, rail fixation application and bone grafting at docking site (*arrow*). **d** Consolidation of callus and union at docking site (just before removal of the system)

In our study, 22 cases underwent segmental transport (14 femur and 8 tibia) and 18 cases were treated with acute docking and bone transport (11 femur and 7 tibia; Fig. 2). Primary bone grafting at docking site was done in 5 cases. Except for the case with the poor result (see above) who refused a secondary grafting procedure to the docking site, none of the other cases required secondary procedures to achieve bony union. Docking site union was noted to be faster when primary bone grafting was done (Figs. 2, 3, 4, 5, 6).

Complications that occurred during the treatment period were graded according to Paley’s working classification as problem, obstacle or true complication [18]. True complications were divided as minor or major. In this study, all patients had one or more pin track infections which resolved with regular pin track dressing and antibiotics (problem). Post-operative joint stiffness resolved completely in 10 patients with physiotherapy. Fifteen patients had pin track infections which required the removal of the pin and or insertion of a new pin (obstacle). True complications presented in the form of joint stiffness. Twenty-two patients had joint stiffness prior to commencement of treatment and showed no improved in range of movement at the end of treatment despite intensive physiotherapy. Two patients who were non-compliant with the post-operative physiotherapy programme developed joint stiffness at the end of treatment period. Eight patients had an axial deformity of greater than 7 degrees. Infection was eradicated in all but 1 patient who refused further treatment. Limb length inequality of greater than 2.5 cm was seen in 10 patients at the end of treatment period. These patients, despite counselling, were non-compliant for the distraction schedule given; they were satisfied with the length achieved and wanted no further distraction. There was one refracture at the docking site after mobilization, and this was managed non-operatively. No patient had premature consolidation, complex regional pain syndrome, persistent oedema or neurovascular complications on completion of treatment.

## Discussion

Treatment for infected non-union improved with the use of the Ilizarov technique and the principle of distraction histogenesis. The principle of tension-stress describes the response of tissue when put under gradual stretch, in certain conditions, leads to generation of new tissue (bone, muscle, tendon, nerve, fascia, vessels, skin and its appendages) [19].

The Ilizarov technique produces an increase in the blood supply of the affected bone through biological stimulation at the corticotomy site, but a meticulous debridement is needed to reduce the infection load. In osteomyelitis, debridement and sequestrectomy produces a bone gap. The use of external fixation in managing infected non-union is established [20, 21]. However, the external fixators, without additional methods, do not solve the bone defect problem. The Ilizarov technique removes the bone defect through distraction at the corticotomy site and consequent new bone formation by intramembranous ossification, thus bridging the bone gap. The Ilizarov fixator is stable mechanically and permits axial compression during physiological loading [22]. This technique is a proven method of managing infected and gap non-unions in the femur and tibia [23–25].

The Ilizarov fixator uses wires and circular rings; this, especially in the proximal femur, leads to significant patient discomfort. When used for long periods in the treatment of infected non-unions, there is poor patient compliance and tolerance. In addition, the circular fixator technique is technically complex and is associated with some complications [18]. The rail fixation system (S. H. Pitkar Orthotools, Pune, India) is a uniplanar, dynamised external fixator system based on the principle of distraction histogenesis. The mechanical stability is provided by the tapered pins and variable placement of sliding clamps. In being uniplanar, it cannot be used easily to correct three-dimensional deformities as with a circular fixator but, conversely, allows easy access for secondary plastic surgical procedures. It is more patient-friendly; compression and distraction across a fracture or osteotomy site is simpler than with a circular external fixator [26].

Knee stiffness is not a complication of fixator application always. This disability may arise from the fracture, soft tissue damage, pre-existing stiffness and the presence of infection [2]. Use of the rail fixation system, especially with bone transport and or limb length equalisation, can produce knee stiffness. It is important that intensive physiotherapy follows application of this fixator in the femur.

The limitations of this study include the absence of a control group which gives comparative results. The heterogeneous group of patients in the study (age 7–70 years and gender with 35 males and 5 females) makes firm conclusions difficult as responses to trauma, infection and subsequent healing differ [27, 28]. The monolateral rail was found to be effective in managing infected gap non-unions of the femur and tibia; simplicity, ease of use with better patient compliance make it a preferred choice over the circular fixator except for complex three-dimensional deformity corrections.
